# Could Hepcidin Be a New Biomarker in Patients with Idiopathic Pulmonary Fibrosis (IPF)?

**DOI:** 10.3390/jcm13226823

**Published:** 2024-11-13

**Authors:** Gulcin Yilmaz, Hikmet Çoban, Nurhan Sarioglu, Fuat Erel, Merve Akış Yılmaz, Mustafa Çolak, Merve Yumrukuz Şenel, Adnan Adil Hismioğulları

**Affiliations:** 1 Department of Pulmonology, Faculty of Medicine, Balıkesir University, 10145 Balıkesir, Türkiye; hikmet.coban@balikesir.edu.tr (H.Ç.); nurhangencer@hotmail.com (N.S.); fuat.erel@balikesir.edu.tr (F.E.); mustafa.colak@balikesir.edu.tr (M.Ç.); merve.senel@balikesir.edu.tr (M.Y.Ş.); 2 Department of Medical Biochemistry, Faculty of Medicine, Balıkesir University, 10145 Balıkesir, Türkiye; merve.akis@balikesir.edu.tr (M.A.Y.); hismiogullari@balikesir.edu.tr (A.A.H.)

**Keywords:** hepcidin, idiopathic pulmonary fibrosis, anemia, inflammation, biomarker, erythropoietin, ferritin, Systemic Immune–Inflammation Index

## Abstract

**Objectives:** Hepcidin is a biomarker produced by hepatocytes in chronic disease anemia and is known to increase during chronic inflammation. This study compares the hepcidin levels in idiopathic pulmonary fibrosis (IPF) patients and controls, evaluating its relationship with anemia and systemic inflammation in IPF patients. **Methods:** This study included 82 IPF patients and 31 controls. Hepcidin levels were compared between the two groups. In the IPF group, the hepcidin and anemia parameters were compared between anemic and non-anemic patients. The significance between the hepcidin and systemic inflammation parameters such as Erythrocyte Sedimentation Rate, CRP (C-reactive protein) levels, ferritin levels, and the Systemic Immune–Inflammation Index (SII) was investigated. Erythrocyte Sedimentation Rate, C-reactive protein (CRP) levels, and ferritin levels were measured using automated analyzers. Hepcidin and erythropoietin (EPO) levels were determined using ELISA kits. **Results:** A significant difference in hepcidin levels was found between the IPF and control groups (37.13 ± 14.92 vs. 25.77 ± 11.25, *p* < 0.001). No significant difference in hepcidin levels was found between anemic and non-anemic IPF patients (38.25 ± 16.2 vs. 36.7 ± 14.6, *p* = 0.719). No significant correlation was found between hepcidin levels and anemia parameters (serum iron, ferritin, vitamin B12, serum transferrin, transferrin saturation, total iron-binding capacity, hemoglobin, folate, and erythropoietin) in IPF patients. Despite significant differences in the systemic inflammation parameters (ferritin and CRP) between patients and controls, no significant correlation was found between their hepcidin and systemic inflammation parameters. **Conclusions:** Our study demonstrates that the hepcidin levels in IPF patients are elevated independently of anemia and systemic inflammation. We propose that hepcidin could be a potential biomarker to be investigated in IPF patients.

## 1. Introduction

Idiopathic pulmonary fibrosis (IPF) is an irreversibly progressive interstitial lung disease characterized by the progressive fibrosis of lung tissue, with an unknown etiology. This condition manifests as a destructive disease that severely impacts patients’ quality of life, resulting in loss of normal lung architecture, as well as dyspnea, dry cough, and oxygen deficiency [1]. As the most severe form of pulmonary fibrosis, the average survival time for patients diagnosed with IPF is less than five years [2,3].

Although the exact etiology of idiopathic pulmonary fibrosis (IPF) remains to be fully understood, environmental exposures such as cigarette smoke, inhalation of wood and metal dust, and factors such as gastroesophageal reflux micro-aspiration have been identified as risk factors. These factors are thought to cause repetitive damage to the lung epithelium in genetically predisposed individuals [4].

Recent studies have proposed that small amounts of damage to the lungs, which accumulate over time due to aging, lead to repair defects in cells and may play a significant role in the pathogenesis of idiopathic pulmonary fibrosis (IPF). Another important hypothesis regarding the pathogenesis of IPF is the theory of autoinflammation. This theory suggests that repeated epithelial injury and the subsequent wound healing processes that develop in response to this injury play a critical role in the progression of the disease [5].

The fibrotic process in idiopathic pulmonary fibrosis (IPF) is characterized by the accumulation of excessive extracellular matrix proteins produced by myofibroblasts. This overproduction and accumulation of proteins exceeds normal wound healing responses, leading to the structural remodeling of lung tissue. The proliferation and differentiation of alveolar type 2 epithelial cells are necessary to replace the damaged epithelium. However, repeated injuries may diminish the regenerative capabilities of the epithelium and induce abnormal activation of fibroblasts, resulting in restricted alveolar regeneration and excessive extracellular matrix accumulation. This situation ultimately leads to permanent damage to lung tissue and loss of function [6,7].

Alterations in iron metabolism in chronic inflammatory diseases have increased the importance of inflammation-related markers. Hepcidin, a peptide hormone responsible for the regulation of iron homeostasis, is increasingly recognized as a significant biomarker in chronic inflammatory conditions.

Hepcidin is a 25 amino acid peptide hormone that was first isolated from human urine and named according to its site of synthesis and its in vitro antibacterial properties.

The human hepcidin gene (HAMP; OMIM 606464) is located on chromosome 19q13.1 and is primarily produced by the liver [8]. However, Chen et al. demonstrated that hepcidin is also expressed in human airway epithelial cells and alveolar macrophages [9].

Studies on chronic obstructive pulmonary disease (COPD) models have shown that hepcidin plays a critical role in alveolar macrophage function [10]. Increasing evidence suggests that hepcidin is becoming an important biomarker [11,12]. These findings indicate that hepcidin may potentially be used as a biomarker in lung diseases.

Hepcidin levels are regulated by three independent primary mechanisms: inflammation, iron overload, and erythropoietic activity. Inflammation and iron overload increase hepcidin production, whereas erythropoietic activity suppresses its production [13,14].

Elevated hepcidin expression can lead to the development of iron deficiency anemia [15]. Transgenic mice with overexpression of hepcidin exhibit iron deficiency anemia [16,17]. Hypoxia is a strong stimulus for EPO production and erythropoiesis, and it has been shown that hepcidin levels decrease following exercise in hypoxic conditions. These findings indicate an inverse relationship between EPO and hepcidin levels [18,19,20].

Biomarkers play a crucial role in identifying the risk of disease development and in early diagnosis, determining prognosis, and monitoring treatment response. Pulmonary fibrosis is often detected in the advanced stages of the disease, when the lungs are severely damaged and no longer function properly. Therefore, reliable and easily detectable markers are needed to identify and monitor the onset and progression of pulmonary fibrosis at the earliest possible stage. The discovery and validation of such markers would greatly facilitate the development of new therapeutic strategies in the management of diseases such as IPF.

Alveolar epithelial markers, such as surfactant protein-D (SP-D), Krebs von den Lungen-6 (KL-6), and mucin 1 (MUC-1), have been used as part of clinical practice for the diagnosis of interstitial lung diseases (ILDs) in Japan for more than a decade. However, clinical studies validating the potential and effectiveness of these markers as biomarkers in idiopathic pulmonary fibrosis (IPF) remain limited [21].

Studies on certain molecules, particularly matrix metalloproteinases (such as MMP-7 and MMP-1) and Krebs von den Lungen-6 (KL-6), suggest that these molecules could be used as biomarkers in idiopathic pulmonary fibrosis (IPF) in the near future. However, to date, no biomarker has been definitively accepted for IPF [22].

To the best of our knowledge, currently, no studies have investigated the relationship between idiopathic pulmonary fibrosis (IPF) and hepcidin. Therefore, this study aims to evaluate the potential role of hepcidin as a biomarker in patients with IPF.

## 2. Methods

### 2.1. Study Design and Subjects

This single-center case–control study was conducted at the Pulmonology Clinic of Balikesir University Health Practice and Research Hospital from November 2023 to August 2024.

This study included 82 IPF patients diagnosed according to ATS/ERS 2022 criteria and who were in a stable period, and 31 control subjects without pulmonary fibrosis. The inclusion criteria for patients with idiopathic pulmonary fibrosis (IPF) were those diagnosed with IPF according to the ATS/ERS 2022 guidelines and in a stable phase of the disease. Exclusion criteria included individuals with acute exacerbations of IPF in the past three months, other infectious diseases, rheumatological disorders, liver and kidney failure, and those with cancer that could lead to systemic inflammation. The control group consisted of 31 individuals over 60 years of age with comorbidities similar to those in the IPF group but without pulmonary fibrosis. The Charlson Comorbidity Index (CCI) was calculated to assess comorbidities for both the IPF and control groups.

Demographic data (age, smoking habits, and comorbidities) for both the IPF and control groups were recorded. All patients with idiopathic pulmonary fibrosis (IPF) underwent pulmonary function tests and carbon monoxide diffusion capacity testing in accordance with the American Thoracic Society (ATS) guidelines at our outpatient clinic. Furthermore, a six-minute walk test was conducted for all patients. All data were meticulously recorded.

IPF patients were additionally stratified into two groups based on anemia status. Anemia was defined as hemoglobin levels <12 g/dL in women and <13 g/dL in men. To evaluate the etiology of anemia, serum ferritin, iron, total iron-binding capacity, transferrin saturation, vitamin B12, folate, and erythropoietin (EPO) levels were measured.

All procedures of this study were conducted in accordance with the Helsinki Declaration and received approval from the Ethical Committee of the Faculty of Medicine at Balıkesir University (Ethics Committee date: 11 October 2023, decision no: 2023/143). Informed consent forms were obtained from all participants included in this study.

### 2.2. Laboratory Assessments

Blood samples for complete blood count and serum analysis were obtained from all participants in the IPF and control groups after an overnight fast. Serum was separated by allowing for the blood collected in yellow-capped gel tubes to clot and subsequently centrifuging the substance at 4000 rpm for 10 min at +4 °C. A portion of the serum was aliquoted into Eppendorf tubes and stored at −40 °C for hepcidin analysis.

Complete blood count was determined in the collected blood samples using the DxH 800 hematology analyzer (Beckman Coulter, Brea, CA, USA). Serum iron and unsaturated iron-binding capacity (UIBC) levels were measured using the AU680 analyzer (Beckman Coulter, Brea, CA, USA). Serum ferritin, vitamin B12, and folate levels were assessed using the UniCel DxI 600 analyzer (Beckman Coulter, Brea, CA, USA).

Serum C-reactive protein (CRP) levels were determined using the BN II analyzer (Siemens Healthineers, Marburg, Germany), while erythrocyte sedimentation rate (ESR) was measured with the ALS-100 analyzer (Alaris, Izmir, Turkey).

Transferrin saturation percentage was calculated using the formula: [TSAT (%) = iron (µmol/L)/TIBC (µmol/L) × 100]. All analyses were conducted in the Biochemistry and Microbiology Laboratory of Balıkesir University Health Practice and Research Hospital.

Serum EPO levels were analyzed at Gelişim Medical Laboratory using the Siemens Immulite 2000 XPi immunoassay system (Siemens Healthcare Diagnostics Inc., Flanders, NJ, USA).

### 2.3. Serum Hepcidin Analysis

Serum hepcidin levels were determined using a commercially available enzyme-linked immunosorbent assay (ELISA) kit (Human Hepcidin ELISA, Elabscience, Cat# E-EL-H6202, Houston, TX, USA).

Hepcidin measurement was conducted spectrophotometrically at a wavelength of 450 nm using a multimode plate reader (Varioskan Flash Multimode Reader, Thermo Scientific, Waltham, MA, USA). The kit has a measurement range of 0.78–50 ng/mL, a sensitivity of 0.32 ng/mL, and a coefficient of variation of less than 10%. All procedures were performed in accordance with the manufacturer’s instructions. Serum hepcidin measurement was conducted in the Medical Biochemistry Laboratory of Balıkesir University Faculty of Medicine.

### 2.4. Statistical Method

Statistical analyses were performed using the SPSS 25.0 software package (SPSS Inc; Chicago, IL, USA). Continuous variables were presented as mean ± SD or median and range (min–max). Variables with normal distribution were expressed as mean ± SD, while those not normally distributed were reported as median and range. An independent samples *t*-test was used for comparing normally distributed parameters, and the Mann–Whitney *U* test was applied for non-normally distributed parameters. A *p*-value of *p* < 0.005 was considered statistically significant, indicating a meaningful difference between the patient and control groups. The relationship between hepcidin levels and other variables was analyzed using Spearman’s correlation coefficient.

## 3. Results

A total of 82 patients diagnosed with idiopathic pulmonary fibrosis (IPF) and 31 individuals without pulmonary fibrosis (control group) were included in this study. The characteristics of the IPF patients and healthy volunteers are presented in Table 1. Hypertension was the most common comorbidity across all study groups, occurring in 34/82 IPF patients and 18/31 healthy individuals. In our study population, diabetes mellitus was observed in 19/82 IPF patients and 13/31 individuals in the control group. The patient and control groups were found to be similar in terms of age and comorbidities.

To investigate the factors that may influence hepcidin expression in IPF patients, we measured peripheral blood hepcidin concentrations along with parameters reflecting erythropoietic activity, iron metabolism, and systemic inflammation.

In terms of erythropoietin (EPO) levels (4.3–29) [median (min–max), mIU/mL], the IPF patients [10.60 (1.78–57.3)] and the control group [6.74 (1.05–59)] were similar (*p* = 0.058). Anemia parameters and EPO levels were also found to be comparable between the two groups.

**Figure 1:**
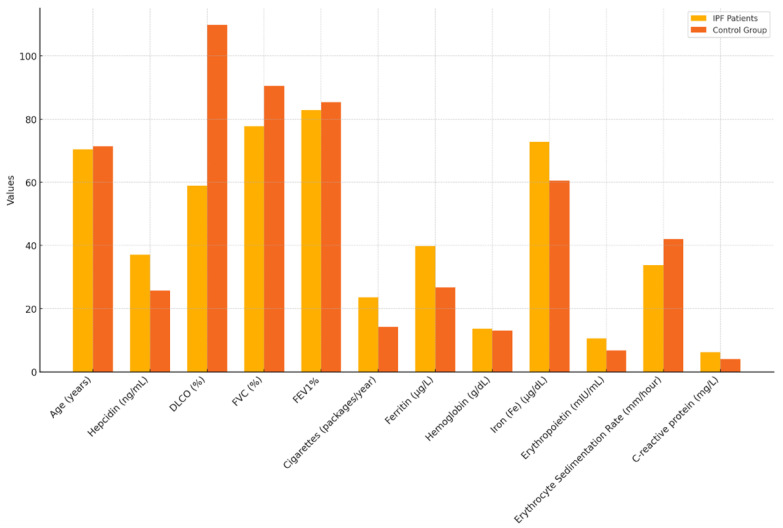
Demographic characteristics of IPF patients and control group.

**Figure 2:**
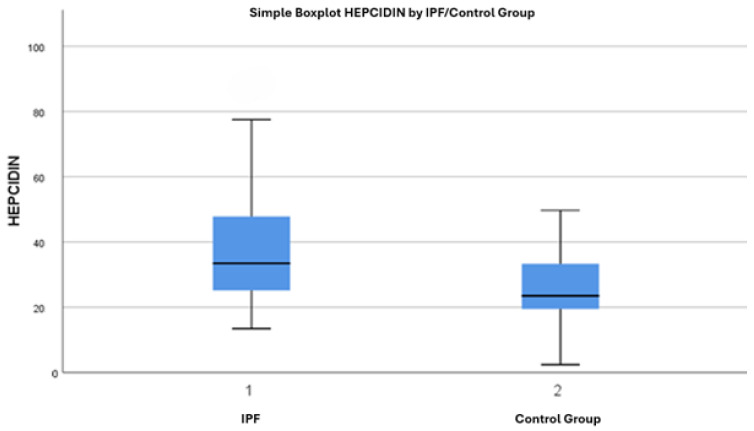
Serum hepcidin levels in IPF patients (1) and control group (2).

Hepcidin concentrations were 37.13 ± 14.92 in IPF patients and 25.77 ± 11.25 in the control group. A significant difference was observed when comparing hepcidin levels between the IPF patients and the control group (*p* < 0.001) (Table 1). Hepcidin levels were found to be higher in the IPF patient group compared to the control group (Figure 1 and Figure 2).

The DLCO (%) Mean ± SD values in IPF patients were 58.95 ± 3 1.937, which were significantly lower compared to the control group (109.83 ± 9.48) (*p* < 0.001).

The FVC% Mean ± SD values were 77.79 ± 19.0 in IPF patients, which were significantly lower than in the control group (90.57 ± 24.94) (*p* = 0.006).

The MMRC score was significantly higher in IPF patients (1.63 ± 1.07) compared to the control group (0.58 ± 0.92) (*p* < 0.001).

The number of cigarettes (packages/year) used, expressed as Mean ± SD, was higher in IPF patients (23.59 ± 23.27) compared to the control group (14.19 ± 22.58) (Table 1).

Ferritin levels [Median (min–max), μg/L] were higher in IPF patients [39.90 (10.5–469)] compared to the control group [26.70 (3.70–266)] (*p* = 0.030).

C-reactive protein (mg/L) levels were higher in IPF patients [6.27 (3–66)] compared to the control group [4.15 (3–9.35)] (*p* = 0.048).

The six-minute walk test distance was significantly lower in IPF patients (366.74 ± 102.50) compared to the control group (472.8 ± 79.0) (*p* = 0.001) (Table 1).

The Systemic Immune–Inflammation Index (SII) was higher in IPF patients (903.43 ± 699.78) compared to the control group (680.21 ± 516.1), although this difference was not statistically significant (*p* = 0.109).

The Erythrocyte Sedimentation Rate (mm/hour) was 33.85 ± 24.01 in IPF patients and 42.06 ± 36.96 in the control group (*p* = 0.169). This difference was also not statistically significant (Table 1).

When comparing IPF patients with the control group, a significant difference was found in the levels of systemic inflammation markers CRP and ferritin, whereas no significant difference was observed in the SII and Erythrocyte Sedimentation Rate parameters (Table 1).

There was no statistically significant difference in hepcidin levels between patients with anemia (*n* = 20), (38.25 ± 16.2) and those without anemia (*n* = 62) (36.7 ± 14.6) in the IPF patient group (*p* = 0.702) (Table 2).

Age was similar between the groups of IPF patients with and without anemia (*p* = 0.23). In IPF patients with anemia, the hemoglobin (Hb) level was 11.07 ± 0.82, while in the non-anemic group, the Hb level was 14.5 ± 1.38.

There was a significant difference in iron levels between the two groups (*p* = 0.02), whereas no significant differences were observed in UIBC, ferritin, and erythropoietin levels (*p* = 0.718, *p* = 0.985, and *p* = 0.181, respectively).

A significant difference was observed when comparing hepcidin levels between IPF patients and the control group, with hepcidin levels found to be higher in the IPF patient group, as shown in Figure 2.

Table 3 presents correlation results between hepcidin levels and anemia parameters in IPF patients. 

The IPF patients (13.71 ± 1.97) and the control group (13.10 ± 5.10) were similar in terms of anemia (*p* = 0.364).

**Figure 3:**
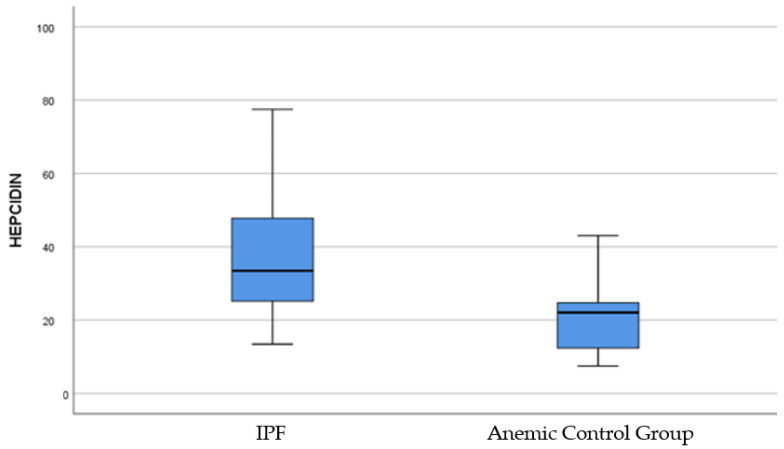
Serum hepcidin levels in IPF patient group and anemic control group.

The hepcidin concentrations of IPF patients (*n* = 82) (37.13 ± 14.92) were found to be higher than those of the control group with anemia (*n* = 10) (22.41 ± 11.47). A significant difference was observed between IPF patients and the anemic control group in terms of hepcidin levels (*p* < 0.003) (Figure 3).

The correlation between hepcidin levels and systemic inflammation parameters in the IPF patient group is presented in Table 4.

No significant correlations were found between serum hepcidin levels and CRP (r = 0.38, *p* = 0.737), ferritin (r = −0.025, *p* = 0.823), Erythrocyte Sedimentation Rate (r = 0.38, *p* = 0.736), or SII (r = 0.164, *p* = 0.141) (Table 4).

## 4. Discussion

The synthesis of hepcidin is regulated by multifactorial influences, including anemia, systemic inflammation, and EPO. Studies in the literature have demonstrated that hepcidin operates independently of iron metabolism in both airway epithelial cells and alveolar macrophages [23,24].

To the best of our knowledge, our study is the first real-world study to investigate serum hepcidin levels in IPF patients. In our study, we demonstrated that hepcidin levels were significantly higher in IPF patients compared to the control group, independent of anemia, EPO, and systemic inflammation. Our findings represent the first study to identify hepcidin as a potentially useful biomarker in IPF.

Alveolar epithelial cells play a central role in the fibroproliferative mechanisms involved in the pathogenesis of IPF [25]. It has been demonstrated that hepcidin is secreted by alveolar epithelial cells in the airways. Frazier et al. showed that airway epithelial cells express hepcidin, and the presence of hepcidin in the airways does not alter cellular iron transport [26].

Mühlfeld et al. demonstrated that disruption of the hepcidin/ferroportin regulatory system affects the structure of the interalveolar septa in mouse lungs, characterized by thickening of the air–blood barrier and hyperplasia and hypotrophy of alveolar type 2 epithelial cells [27]. In our study, serum hepcidin levels were found to be higher in the IPF patient group compared to the control group. This elevation may be indicative of the involvement of the interalveolar septa.

Hepcidin is crucial for iron homeostasis. Hepatic hepcidin synthesis decreases with increased erythropoietic activity, hypoxia, and reduced iron stores. Conversely, hepcidin synthesis increases in the presence of iron overload and systemic inflammation [28].

In a study by Brion et al., the effects of hepcidin 1 deletion on iron metabolism were analyzed by disrupting the hepcidin 1 gene, targeting nearly the entire coding region in mice. In Hepc1(−/−) mice, early and severe multivisceral iron overload developed alongside the preservation of splenic macrophages, with increased serum iron and ferritin levels observed compared to the control group. Mice lacking hepcidin exhibited elevated iron levels [16].

In a study conducted by Nicolas et al., a complete defect in hepcidin expression was shown to be clearly responsible for progressive iron overload in tissues [29]. In a study by Lee et al., it was indicated that genetic mutations in genes such as Tmprss6, resulting in excessive expression of hepcidin, the secretion of hepcidin by hepatic adenomas, and inflammatory diseases that secrete excessive IL-6, can lead to anemia [30]. In mice with hepcidin deficiency, there was an increase in iron levels [16,29]. In contrast, excessive expression of hepcidin has resulted in severe iron deficiency [17,30]. In our study, no significant correlation was identified between hepcidin levels and parameters of iron metabolism and anemia in patients with idiopathic pulmonary fibrosis (IPF).

Hepcidin has been identified as a potential systemic inflammation biomarker in various diseases. Huang et al. demonstrated its relevance in Kawasaki disease [11], while Arabul et al. explored its predictive capacity in severe acute pancreatitis [12]. Leuenberger et al. employed hepcidin to identify autologous blood transfusions [31], and Wagner et al. highlighted its prognostic potential as a biomarker in chronic kidney disease [32,33]. Çelik et al. reported no positive correlation between serum hepcidin levels and inflammatory markers in patients with ulcerative colitis (UC) [34].

In a study by Çiçek et al., which included 25 patients with Behçet’s disease, 30 patients with recurrent aphthous stomatitis (RAS), and 25 healthy controls, the low serum and salivary prohepcidin and hepcidin concentrations observed in RAS and Behçet’s patients suggested a potential role for hepcidin in the etiopathogenesis of these diseases. However, elevated hepcidin levels were not detected in this study conducted on patients with Behçet’s disease, an inflammatory condition [35].

In a study conducted by Ustamanolakis et al. involving 100 patients with inflammatory bowel disease [49 with ulcerative colitis (UC) and 51 with Crohn’s disease (CD)] and 102 healthy controls, hepcidin levels were found to be significantly higher in both UC and CD patients compared to healthy controls (*p* < 0.0001) [36].

In a study by Duru et al., 74 male patients (aged 45–75 years) with stable chronic obstructive pulmonary disease (COPD) were grouped into Group I: mild COPD (*n* = 25), Group II: moderate COPD (*n* = 24), and Group III: severe COPD (*n* = 25). This study found no significant difference in hepcidin levels between the healthy control group and the mild COPD group (*p* = 0.781), whereas significant differences were observed between the moderate (*p* = 0.004) and severe COPD groups (*p* = 0.002). Hepcidin levels in the control group were higher than those in the moderate and severe COPD groups [37].

Studies conducted on patients with ulcerative colitis (UC), Crohn’s disease, and chronic obstructive pulmonary disease (COPD) have demonstrated elevated hepcidin levels compared to healthy controls [38,39]. In our study, hepcidin, CRP, and ferritin levels were significantly elevated in the IPF patient group compared to the control group, while no significant association was observed with Erythrocyte Sedimentation Rate and SII parameters. Moreover, no significant correlation was found between hepcidin and the systemic inflammation markers CRP, ferritin, Erythrocyte Sedimentation Rate, and SII.

Although hepcidin is predominantly produced by hepatocytes, it can also be synthesized in other tissues, including the heart [38,39], kidneys [40], adipose tissue [41], spinal cord [42], myeloid cells [43], spleen and alveolar macrophages [44], monocytes [45], and alveolar epithelial cells [26]. Hepcidin is expressed at lower levels in human airway epithelial cells and alveolar macrophages, suggesting a potential paracrine role in the lungs [9].

Nguyen et al. demonstrated that lipopolysaccharide-stimulated murine alveolar macrophages increased hepcidin mRNA expression, while iron treatment had no effect on hepcidin mRNA expression [23,46]. Frazier et al. showed that hepcidin had no significant effect on iron transport in airway epithelial cells and alveolar macrophages [26]. In our study, no significant correlation was found between hepcidin levels and the parameters of iron metabolism.

Adel et al. demonstrated that the serum hepcidin levels in COPD patients were significantly lower compared to that in the control group and correlated with the severity of COPD and hypoxemia [47]. This decrease was suggested to be secondary to a reduction in alveolar epithelium. In our study, we observed a significant elevation in serum hepcidin levels in the IPF patient group compared to the control group. We hypothesize that this increase may be attributed to an upregulation of hepcidin synthesis in alveolar epithelial cells, likely due to a complex mechanism involving multiple factors. The decrease in hepcidin levels with increasing severity of COPD supports the notion that this is related to a reduction in alveolar epithelial cells.

Fibroblasts and myofibroblasts play critical roles in the fibrosis process of IPF. The excessive production of extracellular matrix proteins by these cells leads to structural changes in the lung tissue. The activation of these cells is essential in the processes of alveolar epithelial cell damage and regeneration. The relationship between this activation and hepcidin is considered to be a potential monitoring tool [48].

Our findings may provide a significant foundation for future research on the use of hepcidin as a biomarker in diagnosing or monitoring disease progression in IPF. Furthermore, these findings suggest that hepcidin may be produced by alveolar epithelium in response to fibrotic stimuli, and could have potential as a marker for determining disease severity and prognosis.

### Study Limitations

The limitations of this study include its single-center design and the small number of IPF patients with anemia, as well as the limited number of anemic individuals in the control group. Therefore, further studies involving larger patient cohorts and multiple centers are warranted.

## 5. Conclusions

Our study is the first to investigate hepcidin levels in IPF. In addition to its systemic functions, hepcidin is synthesized in alveolar epithelial cells and in response to parenchymal damage. The elevated hepcidin levels observed in IPF patients suggest that it may serve as a biomarker of parenchymal injury. Larger-scale studies evaluating clinical symptoms and additional parameters are needed to better define the role of hepcidin in IPF and to establish its potential as a biomarker.

## Figures and Tables

**Table 1:** Comparison of demographic and laboratory characteristics of IPF patients and control group.

	IPF Patients	Control Group	*p*
Age (years) mean ± SD	70.5 ± 7.69	71.42 ± 7.56	0.230
Hepcidin (ng/mL) mean ± SD	37.13 ± 14.92	25.77 ± 11.25	<0.001
DLCO (%) * mean ± SD	58.95 ± 31.937	109.83 ± 9.48	<0.001
FVC% ** mean ± SD	77.79 ± 19.0	90.57 ± 24.94	0.006
FEV_1_% *** mean ± SD	82.87 ± 19.93	85.4 ± 25.76	0.587
mMRC ****	1.63 ± 1.07	0.58 ± 0.92	<0.001
Cigarettes (packages/year) Mean ± SD	23.59 ± 23.27	14.19 ± 22.58	0.054
Serum transferrin (g/L) Mean ± SD	2.72 ± 2.17	3.61 ± 4.27	0.192
Ferritin (18–250 μg/L) Median (min–max)	39.90 (10.5–469)	26.70 (3.70–266)	0.030
Hemoglobin (12–16 gr/dL) Mean ± SD	13.71 ± 1.97	13.10 ± 5.10	0.364
Iron (Fe) (50–170 μg/dL) Mean ± SD	72.83 ± 35.98	60.5 ± 34.29	0.106
Vitamin B12 (ng/L)	203 (83–1500)	198 (77–1166)	0.801
Folate Median (min–max) (μg/L)	6.10 (3.5–21.20)	7.15 (3.3–281)	0.271
UIBC ***** (228–428 μg/dL) Median (min–max)	258.9 ± 69.51	288.46 ± 99.0	0.841
Erythropoietin (4.3–29) Median (min–max) (mIU/mL)	10.60 (1.78–57.3)	6.74 (1.05–59)	0.058
Erythrocyte Sedimentation Rate (mm/hour)	33.85 ± 24.01	42.06 ± 36.96	0.169
C-reactive protein (mg/L)	6.27 (3–66)	4.15 (3–9.35)	0.048
Six-minute walk test	366.74 ± 102.50	472.8 ± 79.0	<0.001
SII ******	903.43 ± 699.78	680.21 ± 516.1	0.109
CCI ******* score	3.52 ± 1.740	3.58 ± 1.23	0.856
Treatment duration (months)	16.75 ± 17.60	-	-
Follow-up duration (months)	16.75 ± 17.6	-	-

* DLCO: Diffusing capacity for carbon monoxide, ** FVC: Forced Vital Capacity, *** FEV1%: Forced Expiratory Volume in 1 s, **** mMRC: Modified Medical Research Council; ***** UIBC: unsaturated iron-binding capacity. ****** SII: Systemic Immune–Inflammation Index; ******* CCI: Charlson Comorbidity Index.

**Table 2:** This table presents a comparison of hepcidin levels and anemia-related parameters (ferritin, hemoglobin, iron, UIBC, erythropoietin) between patients with and without anemia.

	İPF		
	Anemic	Non-Anemic	*p*
Age (years) Mean ± SD	70.65 ± 7.33	70.30 ± 7.27	0.230
Hepcidin (ng/mL) Mean ± SD	38.25 ± 16.2	36.7 ± 14.6	0.702
Ferritin (μg/L) Median (min–max)	30.80 (3.70–469)	40.60 (8.10–329)	0.985
Hemoglobin (12–16 gr/dL) Mean ± SD	11.07 ± 0.82	14.5 ± 1.38	0.000
Iron (Fe) (50–170 ug/dL) Mean ± SD	50.05 ± 26.3	79.7 ± 35.8	0.02
UIBC * (228–428 μg/dL) Mean ± SD	253.72 ± 71.2	260 ± 69.5	0.718
Eritropoietin median (mIU/mL) (min–max)	13.95 (1.78–99.20)	9.18 (1.05–76.90)	0.181
Vitamin B12 (min–max)	250 (84–1500)	172 (83–797)	0.213
Folate	6.40 (2.40–12)	6.5 (2.40–23.30)	0.928

* UIBC: unsaturated iron-binding capacity.

**Table 3:** Correlation between hepcidin levels and anemia parameters in IPF patients.

Hepcidin (ng/mL)	r	*p*
UIBC * (μg/dL)	0.64	0.587
Vitamin B12 (ng/L)	0.123	0.283
Iron (μg/dL)	−0.105	0.365
Ferritin (μg/L)	−0.025	0.823
Folate (μg/L)	0.040	0.732
Hemoglobin (g/dL)	0.052	0.643
Transferrin saturation (%)	−0.085	0.467
Serum transferrin (g/L)	0.054	0.654
Erythropoietin (mIU/mL)	0.197	0.099

* UIBC: unsaturated iron-binding capacity.

**Table 4:** Correlation between hepcidin and systemic inflammation parameters in the IPF patient group (*n* = 82).

Hepcidin	r	*p*
SII *	0.16	0.141
Ferritin (μg/L)	−0.025	0.823
C-reactive protein (mg/L)	0.38	0.737
Erythrocyte Sedimentation Rate (mm/hour)	0.38	0.736

* SII: Systemic Immune–Inflammation Index.

## Data Availability

The data can be obtained from the corresponding author upon reasonable request.
